# 

**Published:** 1889-10

**Authors:** 


### Ohio Valley Dental Society.

The eighth quarterly meeting of the Ohio Valley Dental Society, will be held at the office of Dr. J. E. Barricklow, Flushing, Ohio, on Monday, October 7th, 1889, at 7 p. m.

Come prepared to take part in an interesting meeting.

PROGRAMME.

*ESSAY.*—"What shall be done with the Third Molar?"
By Dr. J. G. Parr.

*ESSAY.*—"Extraction,"
By Dr. F. E. Battershell.

*ESSAY.*—"How to conduct a practice."
By Dr. Chas. E. Mason.

Incidents of Office Practice.

Question Box.

Each member is invited to bring some appliance to present before the Society. F. S. Maxwell, Sec'y.

### Ohio State Society.

The annual meeting of this body will be held at the Hollenden Hotel, in Cleveland, on Tuesday, Oct. 29th, and continue three days. A good programme is presented and we are assured that a goodly number of papers have already been promised. A large and interesting meeting is anticipated. This is the first time this society has met in Cleveland, and the energy and enthusiasm of the profession in northern Ohio gives large promise for this meeting.

The membership of societies in neighboring States will remember that they have a standing invitation to be present at these annual convocations, and take part in the proceedings; and it is to be hoped that there will be a large number of acceptances on this occasion.

### Fifth Annual Meeting.

The Ohio State Dental Society (reorganized) will meet in Cleveland, Oct. 29th, 30th, 31st, 1889, at "The Hollenden," corner Superior and Bond streets. Sessions commense at 10 A. M. Oct. 29th.

PROGRAMME.

1. Address by...............PRES. C. M. WRIGHT, Cincinnati.
2. Paper.—"Dental Education."..........J. TAFT, Cincinnati.
3. Paper.—"Failures."..............C. R. BUTLER, Cleveland.
4. Paper.—"Legal Liability of Corporations for Injuries the Result of Their Negligence."............H. A. SMITH, Cincinnati.
5. Paper.—"Excelsior."H. H. HARRISON, Wheeling, W. Va.
6. Paper.—"Amblyopia."........D. R. JENNINGS, Cleveland.
7. Paper.—"One way of Increasing Dentists Usefulness." ........................................H. M. KEMPTON, Cincinnati.
8. Paper.—................"Woman's Work in the Profession." ...................................MARTHA J. ROBINSON, Cleveland.
9. Report of Pres. of 7th Dist. Society.DR. E. G. BETTY, Cin.

There will several clinics, but by whom or of just what nature, the committee will not be able to announce until the time of meeting.

The committee may alter the arrangement of papers at time of meeting to suit writers of papers. J. E. ROBINSON,

Chairman Committee of Arrangements.

### Board of Dental Examiners Meeting.

The annual meeting of the Ohio State Board of Dental Examiners will be held in Cleveland on the 29th and 30th of October. The first meeting will be held at 12 o'clock on Tuesday the 29th, in the parlors of the Hollenden Hotel. All interested will please make a note of this and govern themselves accordingly.

The tenth International Medical Congress to be held at Berlin will be opened August 4, 1890. The proceedings will terminate on August 9. A general meeting of representatives of all the medical faculties and societies in Germany has been called by Professors Virchow, von Bergmann, and Waldeyer. It will meet at Heidelberg on September 17, to discuss the arrangements to be made for the congress. Among those expected to be present at this conference are many well-known medical men of Germany. Among other names are those of von Ziemssen, Biermer, Czerny, von Zehender, Schwalbe, Lichtheim, Weigert, Trendelenburg, Mosler, Binz, Hegar, Leichtenstern, von Zenker, Schonborn, Naunyn, Graf, His, and Hitzig.

### For Sale.

A Dental Office which is announced in the advertising pages of this number of the Register, is eminently worthy of the attention of any good and experienced dentist, who may desire a good location and an old and well established practice and office.

It is in a wealthy community, and a city of over eleven thousand inhabitants. All the instruments are for sale, and the office can be had for a series of years.

### CONTENTS.

COMMUNICATIONS.

A MONTHLY MAGAZINE DEVOTED TO THE INTERESTS OF THE

**DENTAL PROFESSION.**

*Terms Reduced to* $2.00 *Per Year. Single Copies,* 25 *Cents.*

**EDITED BY PROF. J. TAFT, D.D.S.**

—AND—

**Published by SAM'L A. CROCKER & CO., Ohio Dental and Surgical Depot.**

The volume commences in January and closes in December.

The REGISTER being issued monthly is always fresh, therefore Indispensable to the practitioner who desires to keep posted up with the new inventions and improvements which are daily developed. Neither expense nor effort will be spared to give value and variety to its contents. Articles will be illustrated with well executed engravings whenever they will add to the interest or assist to explanation.

Its extensive circulation and frequency of issue make it one of the BEST DENTAL ADVERTISING MEDIUMS in the United States.

All subscriptions must begin with January or July.

Local or special notices, five lines or less, will be inserted for $3 each insertion; over five lines, 50 cts. per line.

All advertising bills payable in advance, or at furthest, after the issue of the first number containing the advertisement. All transient advertisements to be paid for in advance.

☞CONTRIBUTIONS SOLICITED.

Exclusively Editorial Communications should be addressed to the Editor.

The latest number of the REGISTER may be ordered with or without other goods at any time, and all letters should be addressed to

SAM'L A. CROCKER & CO., Ohio Dental and Surgical Depot, Cincinnati, O.

